# Supplementary material for: Physical, sexual and psychological intimate partner violence and non-partner sexual violence against women and girls: a systematic review protocol for producing global, regional and country estimates
Source: BMJ Open. 2021 Aug 19;11(8):e045574. doi: 10.1136/bmjopen-2020-045574 (PMC8378393; doi:10.1136/bmjopen-2020-045574)
Supplement: Author's manuscript [file bmjopen-2020-045574.draft_revisions.pdf]

BMJ Open is committed to open peer review. As part of this commitment we make the peer review history of every article we publish publicly available.

When an article is published we post the peer reviewers' comments and the authors' responses online. We also post the versions of the paper that were used during peer review. These are the versions that the peer review comments apply to.

The versions of the paper that follow are the versions that were submitted during the peer review process. They are not the versions of record or the final published versions. They should not be cited or distributed as the published version of this manuscript.

BMJ Open is an open access journal and the full, final, typeset and author-corrected version of record of the manuscript is available on our site with no access controls, subscription charges or pay-per-view fees (http://bmjopen.bmj.com).

If you have any questions on BMJ Open's open peer review process please email info.bmjopen@bmj.com

BMJ Open

Physical, sexual and psychological intimate partner violence and non-partner sexual violence against women and girls: a systematic review protocol for producing global, regional and country estimates

Journal:	*BMJ Open*
Manuscript ID	bmjopen-2020-045574
Article Type:	Protocol
Date Submitted by the Author:	06-Oct-2020
Complete List of Authors:	Stoeckl, Heidi; LSHTM, Global Health and Development<br>Sardinha, Lynnmarie; World Health Organization, Department of Reproductive Health and Research<br>Meyer, Sarah; World Health Organization, Department of Sexual and Reproductive Health and Research<br>Maheu-Giroux, Mathieu; McGill University, Epidemiology and Biostatistics<br>Garcia-Moreno, Claudia; World Health Organization
Keywords:	PUBLIC HEALTH, EPIDEMIOLOGY, STATISTICS & RESEARCH METHODS

SCHOLARONE™
Manuscripts

BMJ

I, the Submitting Author has the right to grant and does grant on behalf of all authors of the Work (as defined in the below author licence), an exclusive licence and/or a non-exclusive licence for contributions from authors who are: i) UK Crown employees; ii) where BMJ has agreed a CC-BY licence shall apply, and/or iii) in accordance with the terms applicable for US Federal Government officers or employees acting as part of their official duties; on a worldwide, perpetual, irrevocable, royalty-free basis to BMJ Publishing Group Ltd ("BMJ") its licensees and where the relevant Journal is co-owned by BMJ to the co-owners of the Journal, to publish the Work in this journal and any other BMJ products and to exploit all rights, as set out in our licence.

The Submitting Author accepts and understands that any supply made under these terms is made by BMJ to the Submitting Author unless you are acting as an employee on behalf of your employer or a postgraduate student of an affiliated institution which is paying any applicable article publishing charge ("APC") for Open Access articles. Where the Submitting Author wishes to make the Work available on an Open Access basis (and intends to pay the relevant APC), the terms of reuse of such Open Access shall be governed by a Creative Commons licence – details of these licences and which Creative Commons licence will apply to this Work are set out in our licence referred to above.

Other than as permitted in any relevant BMJ Author's Self Archiving Policies, I confirm this Work has not been accepted for publication elsewhere, is not being considered for publication elsewhere and does not duplicate material already published. I confirm all authors consent to publication of this Work and authorise the granting of this licence.

For peer review only

Physical, sexual and psychological intimate partner violence and non-partner sexual violence against women and girls: a systematic review protocol for producing global, regional and country estimates

Heidi Stöckl, Gender Violence & Health Centre, London School of Hygiene and Tropical Medicine, London, UK. Heidi.stoeckl@lshtm.ac.uk

LynnMarie Sardinha, Department of Reproductive Health and Research, World Health Organization, Geneva, Switzerland. sardinial@who.int

Sarah Meyer, Department of Reproductive Health and Research, World Health Organization, Geneva, Switzerland. smeyer@who.int

Mathieu Maheu-Giroux, Department of Epidemiology, Biostatistics, and Occupational Health, School of Population and Global Health, McGill University, Montréal, Canada. mathieu.maheu-giroux@mcgill.ca

Claudia García Moreno*, Department of Reproductive Health and Research, World Health Organization, Avenue Appia 20, 1211 Geneva, Geneva, Switzerland. garciamorenoc@who.int

* corresponding author

For peer review only

Abstract

Introduction: In 2013, the World Health Organisation (WHO) published the first global and regional estimates on physical and sexual intimate partner violence (IPV) and non-partner sexual violence (NPSV) based on a systematic review of population-based prevalence studies. In this protocol, we describe a new systematic review for the production of updated estimates for IPV and NPSV for global monitoring of violence against women, including providing the baseline for measuring Sustainable Development Goal to eliminate all forms of violence against women and girls.

Methods and analysis: The systematic review will update and extend the previous search for population-based surveys (either nationally or sub-nationally representative) conducted among women aged 15+ years that measured the prevalence of physical, sexual, psychological and physical and/or sexual IPV, NPSV or sexual violence by any perpetrator up to December 2019. Data will be extracted separately for all age groups, setting (urban/rural), partnership status (currently partnered/ever-partnered/all women), and recall period (lifetime prevalence/past 12 months; for IPV). Studies will be identified from electronic searches of online databases of EMBASE, MEDLINE, Global Health and PsycInfo. A search of national statistics office homepages will be conducted for each country to identify reports on population-based, national or sub-national studies that include data on IPV or NPSV published outside academic journals. Two reviewers will be involved in quality assessment and data extraction of the review. The review is planned to be updated on a continuous basis. All findings will undergo a country consultation process. It has been registered at Prospero: CRD42017054100

Ethics and dissemination: Formal ethical approval is not required, as primary data will not be collected. This systematic review will provide a basis and a follow-up tool for global monitoring of the Sustainable Development Goal Target 5.2 on the elimination of all forms of violence against women and girls.

Strength and limitations

- Gold-standard systematic review process followed by country consultation process to verify global and country estimates on intimate partner violence and non-partner sexual violence
- Multiple international technical advisory meetings to discuss the search procedures, data extraction and analysis plans of the estimates
- Consensus on definitions and comparable measurements of physical and intimate partner violence
- Challenges in the comparability of data on non-partner sexual violence measurements due to a lack of global definitions and measurement tools

Keywords: Violence against women, intimate partner violence, spousal violence, non-partner sexual violence, sexual violence, psychological abuse, psychological partner violence, systematic review, prevalence

For peer review only - http://bmjopen.bmj.com/site/about/guidelines.xhtml

Physical, sexual and psychological intimate partner violence and non-partner sexual violence against women and girls: a systematic review protocol for producing global, regional and country estimates

Background

Intimate partner violence and non-partner sexual violence are well recognized as human rights violations and public health problems [1]. In 2013, the World Health Organisation, the London School of Hygiene and Tropical Medicine, and the South African Medical Research Council conducted three systematic reviews to estimate the worldwide prevalence of intimate partner violence, non-partner sexual violence and intimate partner homicide. They estimated that one in three women experienced physical and/or sexual intimate partner violence or non-partner sexual violence in their lifetime; among all women murdered, 38.6 percent were murdered by their intimate partner [2-5]. The review further established associations between intimate partner violence and numerous adverse health outcomes, finding that women experiencing intimate partner violence were twice as likely to report depressive symptoms, nearly twice as likely to have alcohol use disorders, 16% more likely to have a low birthweight baby, and 1.5 times more likely to acquire HIV or another STI, compared to women who had not experienced intimate partner violence [3]. Similar associations were found for non-partner sexual violence and depression and alcohol use disorders [3, 5].

The growing recognition of the high prevalence and significant health and other impacts of intimate partner violence and non-partner sexual violence has contributed to the inclusion of 'the elimination of all forms of violence against women' in the 2030 Agenda for Sustainable Development. The Sustainable Development Goal (SDG) Target 5.2 on the elimination of all forms of violence against all women and girls in the public and private spheres, is monitored using the following indicators:

> 5.2.1: Proportion of ever-partnered women and girls aged 15 years and older subjected to physical, sexual or psychological violence by a current or former intimate partner in the previous 12 months, by form of violence and by age

> 5.2.2: Proportion of women and girls aged 15 years and older subjected to sexual violence by persons other than an intimate partner in the previous 12 months, by age and place of occurrence.

This article presents the protocol of the systematic review to generate the baseline data for producing estimates that will contribute to the global monitoring of violence against women, including for the SDG process.

Methods/Design

This review builds on and updates the methodology used for the previous systematic reviews on the global prevalence of intimate partner violence and non-partner sexual violence. It will be expanded to include evidence from studies directly received from national statistics offices, the Demographic and Health Surveys and those identified through additional internet searches. A data extraction form and quality assessment form used for the 2013 prevalence review data extraction to collate data from selected studies will also be adapted and expanded to include psychological intimate partner

For peer review only - http://bmjopen.bmj.com/site/about/guidelines.xhtml

violence, non-partner sexual violence and sexual violence by any partner. The Preferred Reporting Items for Systematic Review and Meta-Analysis (PRISMA) guidelines are used as the template for reporting the present review. For the present protocol, the PRISMA statement for Protocols (PRISMA-P) was used for its reporting (see online Appendix 1) [6]. This review was registered in the PROSPERO International Prospective Register of systematic reviews, registration number CRD42017054100 on the 2nd of January 2017.

Aims of the review

To collect all available data on the prevalence of physical and/or sexual intimate partner violence, psychological violence by an intimate partner, non-partner sexual violence and sexual violence by anyone, which can be used to measure advances in addressing violence against women and girls.

Specific review questions are:

1. What is the prevalence of physical and/or sexual intimate partner violence in women 15 years and older, lifetime and in the last 12 months?
2. What is the prevalence of physical intimate partner violence only, lifetime and in the last 12 months?
3. What is the prevalence of sexual intimate partner violence only, lifetime and in the last 12 months?
4. What is the prevalence of psychological intimate partner violence, lifetime and in the last 12 months and how was it measured?
5. What is the prevalence of sexual violence by perpetrator(s) other than a partner only, lifetime and in the last 12 months?
6. What is the prevalence of sexual violence by any perpetrator, lifetime and in the last 12 months?
7. How does the prevalence of intimate partner violence and non-partner sexual violence vary by age group, rural and urban settings over time, lifetime and in the last 12 months?

Criteria for considering studies for this review

Inclusion

- Type of studies: Nationally or sub-nationally representative population-based studies (cross-sectional or cohort studies).
- Type of participants: Studies of women aged 15+.
- Type of outcome: Studies measuring the prevalence of psychological, physical, sexual and physical and/or sexual intimate partner violence, non-partner sexual violence and/or sexual violence by any perpetrator (or studies providing enough data to allow computing these estimates if not directly calculated or reported).
- Only acts based measures of psychological, physical and/or sexual intimate partner violence, non-partner violence and sexual violence by any partner will be included. The authors note that there is convergence on the definitions and standardized measures of physical and sexual intimate partner violence across the world. Psychological intimate partner violence and non-partner sexual violence, however, are less well defined internationally and this systematic review therefore uses the authors' definition when extracting these data and note the different definitions used and the diversity of acts covered to define them.

For peer review only - http://bmjopen.bmj.com/site/about/guidelines.xhtml

Exclusion

- Type of studies: case reports, case series, letters, reviews, policy reports, commentaries, editorials, and administrative data including police statistics on reported crimes
- Types of participants: studies in subgroups of participants that might not be generalizable to the whole population, such as clinical, school or prison samples or studies among pregnant women, same sex partners, or police statistics of reported crimes.
- Outcome type: Estimates that combine prevalence of physical and/or sexual intimate partner violence with other forms of violence, e.g. psychological abuse or studies that did not use a "gold-standard" measure of intimate partner violence defined here as measures asking about a range of specific acts of experience of intimate partner violence because these avoid participants' subjective classification of experiences as "violence" or not.
- Minimum reporting criteria: Studies not giving sufficient detail for extrapolation or imputation, e.g. concrete sample size, confidence intervals or standard error.
- Duplicate reports and/or publications of the same data: the less comprehensive/complete and up-to-date version will be excluded if the same data is reported on. Studies of different years or from different regions identified will all be captured.
- Results will only be extracted if they are representative for a single country, province or town or a geographically restricted area within a country.

Search strategy for identifying relevant studies

A comprehensive and exhaustive search of the Ovid based databases Medline, Embase, Global Health and PsychInfo will be conducted on a regular basis, capturing the years 1989 to 2018 and thereafter to identify all relevant articles that contain data on the prevalence of psychological, physical and/or sexual intimate partner violence and non-partner sexual violence and sexual violence by any perpetrator regardless of the language of publication. The search strategy of relevant terms is detailed in Table 1. Both text words and medical subject heading terms will be used. This is a global study. Any study that is population-based and fits the inclusion criteria will be included and no restrictions in terms of country names or languages will be applied.

Searching of other data sources

Due to the increasing number of violence against women surveys conducted by local and national governments and the regular waves of the Demographic and Health Surveys with a dedicated module on 'domestic violence' which includes intimate partner violence and non-partner sexual violence, manual searches will also be conducted in DHS and other survey reports in a consistent manner in addition to signing up for the regular DHS updates. We will search the webpages of governmental statistical and/or other offices of each individual country for reports that include data on the prevalence of intimate partner violence or non-partner sexual violence. Each report will be traced back to its source to ensure they meet the inclusion criteria, e.g national representative survey on violence against women. Data would be excluded if it is administrative data, including police statistics on reported crimes, as these represent only the sub-set of few women who formally report violence and therefore are known to greatly underestimate the 'true' population prevalence.

Selection of studies for inclusion in the review

Two independent reviewers will use Endnote X7 to independently identify articles and sequentially screen their titles and abstracts for eligibility. Full-texts of articles deemed potentially eligible will be

For peer review only - http://bmjopen.bmj.com/site/about/guidelines.xhtml

retrieved. Further, two independent reviewers will independently assess the full-text of each study for eligibility, and consensually retain studies to be included. Dr García-Moreno will resolve disagreements. A screening guide clearly stating the inclusion and exclusion criteria will be used to ensure that the selection criteria are reliably applied by two review authors. Eligible studies in languages other than English or French will be translated using native speakers or, if unavailable, Google Translate and considered for inclusion.

Data extraction and management

Data will be extracted into an excel sheet independently by two individual researchers and divergences will be checked by a third extractor. In addition, 20% of the data entries will be checked by a third reviewer. Data extraction will furthermore be checked through cross-tabulation of similar fields before data analysis. Data extraction fields will include:

1. Study identifier: Study title, author and publication year
2. Geographical information: Region, country and iso3 codes.
3. Study characteristics: year of beginning of data collection and year of end of data collection, study design (cross-sectional, longitudinal), setting (urban, rural, sub-national, national).
4. Population characteristics: Denominator information (All women sampled, ever-partnered/ ever-married women or currently partnered/currently-married women), and age range of the sample.
5. Information on violence underlying indicator: type of violence (physical, sexual, emotional/psychological, physical and/or sexual), type of violence by perpetrator (physical intimate partner violence, sexual intimate partner violence, psychological intimate partner violence, physical and/or sexual intimate partner violence, sexual violence non-partner, any sexual violence),
6. Who was asked about violence (all women versus ever partnered vs currently partnered) , asked about intimate partner violence by current partner (current partner only or any partner), asked about violence by spouse (violence by spouse only versus violence by a spouse or partner), timing of violence (past year, past two years, ever), severe physical or sexual violence or not (definition by study).
7. Estimate: prevalence point estimate of specific form of violence, type of confidence interval, lower and upper confidence interval, standard error, denominator, numerator.
8. Key quality indicators: Study specified interviewer training on administering questions on violence against women, specialized violence against women survey or a module in a larger survey and whether study was national or subnational.

The quality of studies will be based on the following criteria, and these will be used as adjustment factors in the proposed analyses:

1. whether the study was national or sub-national, because prevalence may differ in areas within a country
2. the type and time of violence measured (whether intimate partner violence was operationalised as physical only, sexual only, past-year only, or severe only) because the prevalence of these forms of violence is lower than the combined lifetime experience of physical and/or sexual intimate partner violence.
3. Whether studies reported that interviewers were trained to ask about violence in a sensitive way as this increases participants willingness to disclose
4. whether studies were specifically designed to measure intimate partner violence or sexual violence prevalence

For peer review only - http://bmjopen.bmj.com/site/about/guidelines.xhtml

For peer review only

5. if studies included only currently-partnered women versus ever-partnered women, as estimates of intimate partner violence exposure in currently partnered women are likely to be lower than in ever-partnered women, particularly for lifetime prevalence, as they would not include women who have been in previously abusive relationships but not in a relationship at the time of the survey
6. if questions pertained to violence from the current partner and/or most recent partner only or from any partner.
7. if questions pertained to violence from a spouse only or from any intimate partner.

Proposed data analysis

The data from this systematic review will be used to estimate the national, global and regional prevalence of physical and/or sexual intimate partner violence, in the past 12 months and lifetime for women aged 15 years and above. For non-partner sexual violence and any sexual violence only global and regional estimates for lifetime prevalence will be produced.

For the pre-processing stage, in what we assume will be rare instances, where study's authors do not report information on the survey sample size, it will be estimated from the standard error or confidence intervals. If both are missing we will conservatively impute sample sizes corresponding to the lowest tercile of the overall distribution of survey samples sizes. If the overall survey sample size of a specific study is available but not the age-specific denominators of the prevalence estimates, we will impute them by distributing this overall sample size proportionally to the age-specific size of the female population reported in the *United Nations World Population Prospect [7]*.

For the analysis stage we will use a Bayesian meta-regression framework. This multilevel modelling approach will use survey-, country-, and region-specific random effects to pool observations from different sources and improve accuracy of estimates by drawing on information from across units. Regions will be defined based on the *Global Burden of Diseases* (GBD) classification. Both nationally and sub-nationally representative studies will be included. We will assume for the latter that they provide estimates that they could inherently be more variable than nationally representative studies. Additionally, this Bayesian regression model will consider heterogeneous age groups (using an age-standardizing approach), account for country-specific age and time trends (using splines), and adjust for key survey differences (denominator, type of violence, etc. – depending on the outcomes) through covariate modelling (with the adjustment calculated outside of the main model to avoid compositional bias). A joint model will be estimated for lifetime and past-year intimate partner violence, imposing the constraint that all age-specific past-year intimate partner violence estimates (for a particular country, at a particular time) should be lower or equal to those estimated for lifetime intimate partner violence.

Patient and public involvement

No patient involved.

Discussion

This systematic review will provide critical data for the global monitoring of progress to eliminate violence against women. It will help to track the effectiveness of efforts made by governments over the coming decade in preventing and addressing intimate partner violence and non-partner sexual violence. The findings will directly feed into the global monitoring, including of the SDG Target 5.2 indicators.

For peer review only - http://bmjopen.bmj.com/site/about/guidelines.xhtml

Over the past years, governments have made commitments to increase the collection of data and research on violence against women, for example in the WHO Global Plan of Action [8], to strengthen the role of the health system in addressing violence, in particular against women and against children. This includes conducting population-based studies to measure the prevalence and nature of violence against women, in particular intimate partner violence and sexual violence by non-partners. This is contributing to an expanding and robust evidence base that will allow, tracking of changes over time. This systematic review will also contribute to the identification of challenges with the instruments and measures being used, especially for the forms of violence for which is there is less international agreement on measurement - psychological intimate partner violence and non-partner violence. It will also identify issues with how data is being reported.

The goal of this review and its future iterations is to contribute key epidemiological information on the magnitude and burden of violence faced by women around the world and how these prevalence patterns may differ by country, region, age, and rural/urban contexts. These data should inform the development and implementation of effective policies and programmes to prevent and respond to violence against women.

The estimates based on the findings of this study will be used by governments, published in WHO/UN reports and as scientific papers in a peer-reviewed journals, and disseminated at relevant fora. The review will be updated regularly and the estimates will be updated approximately every five years to continue to monitor progress made in addressing violence against women.

List of abbreviations

PRISMA: Preferred Reporting Items for Systematic Review and Meta-Analysis

TAG: Technical Advisory Group

For peer review only - http://bmjopen.bmj.com/site/about/guidelines.xhtml

Review status

Data extraction is ongoing as several key data points are still missing, especially on non-partner sexual violence. Existing extracted data is also currently undergoing a country consultation process, which is also adding new estimates. The methods for data analysis and for elaboration of the estimates have been tested and discussed by a technical advisory group (TAG) established by WHO and composed of independent experts. The TAG advises the UN Inter-agency Working Group on VAW data and estimates convened by WHO.

Declarations

Consent for publication: We have mostly used data publicly available or received consent for publication from national Statistical Offices for data missing in reports.

Ethics approval and consent to participate

Not applicable

Availability of data and materials

The datasets generated and/or analysed during the current study will be made available by the WHO on reasonable request.

Competing interests

The authors declare that they have no competing interests

Funding

This work received funding from the UNDP-UNFPA-UNICEF-WHO-World Bank Special Programme of Research, Development and Research Training in Human Reproduction (HRP), a cosponsored programme executed by the World Health Organization (WHO). It also received funding from the Department for international Development, through the UNWomen-WHO Joint Programme on Strengthening Data Collection. The funder had no role in the design of the review.

Authors' contributions

Heidi Stöckl adapted the original search strategy and study design to align it with the discussions on global monitoring of VAW, including measurement of the indicators related to SDG Target 5.2. and developed the inclusion of psychological abuse and any sexual violence into the search and analysis, expanded the search strategy to capture data and reports outside the peer-reviewed literature, participated in deciding on the analysis strategy, and drafted the current manuscript.

LynnMarie Sardinha inputted into the adapted search strategy, study design and data extraction sheet, refined inclusion and exclusion criteria and quality indicators and has been involved in all decisions regarding the current search protocol and this manuscript.

For peer review only - http://bmjopen.bmj.com/site/about/guidelines.xhtml

Mathieu Maheu-Giroux contributed to the establishment the list of key covariates and study characteristics to collect, methods to check the database for integrity and consistency, and conceived the data analysis plan.

Sarah Rachel Meyer inputted into the adapted search strategy, study design and data extraction sheet, refined inclusion and exclusion criteria and quality indicators and has been involved in all decisions regarding the current search protocol and this manuscript.

Claudia García-Moreno co-conceived the original search strategy and study design, inputted into the data extraction sheet, inclusion and exclusion criteria and quality indicators, has been involved in all decisions regarding the current search protocol and this manuscript and provides technical oversight to the whole project. She is the guarantor of the review.

All authors read and approved the final manuscript.

Acknowledgements

This database updates and builds on an earlier similar effort for the WHO, 2013 estimates on the prevalence of IPV and NPSV which was led by Charlotte Watts at the LSHTM and Claudia García-Moreno of WHO. Karen Devries designed and oversaw the earlier systematic searches for intimate partner violence and Naeema Abrahams of the South African Medical Research Council for non-partner violence.

Authors should obtain permission to acknowledge from all those mentioned in the Acknowledgements section.

ew only

For peer review only - http://bmjopen.bmj.com/site/about/guidelines.xhtml

Table 1: Search terms and strategy for the Ovid based databases Medline, PsychInfo, Embase and Global Health

1. meta.ab.
2. synthesis.ab.
3. literature.ab.
4. published.ab.
5. extraction.ab.
6. search.ab.
7. medline.ab.
8. selection.ab.
9. sources.ab.
10. trials.ab.
11. review.ab.
12. articles.ab.
13. reviewed.ab.
14. english.ab.
15. language.ab.
16. randomized.hw.
17. trials.hw.
18. controlled.hw.
19. meta-analysis.pt.
20. review.pt.
21. or/1–20
22. epidemiologic studies/
23. exp case control studies/
24. exp cohort studies/
25. case control.tw.
26. (cohort adj (study or studies)).tw.
27. cohort analy$.tw.
28. (follow up adj (study or studies)).tw.
29. (observational adj (study or studies)).tw.
30. longitudinal.tw.
31. retrospective.tw.
32. cross sectional studies.tw.
33. cross sectional studies/
34. or/22–33
35. Animals/
36. Humans/
37. 35 not (35 and 36)
38. comment.pt.
39. letter.pt.
40. editorial.pt.
41. or/37–40
42. domestic violence/ or partner violence/ or spouse abuse/ or spouse violence/ or domestic abuse/ or partner abuse.mp. [mp = title, original title, abstract, name of substance word, subject heading word]
43. *battered women/
44. (intimate adj4 partner adj4 violence).tw.
45. (intimate adj4 partner adj4 abuse).tw.
46. (intimate adj4 partner adj4 victimi*).tw.
47. domestic abuse.tw.
48. spou$ abuse.tw.
49. dating violence.tw.

For peer review only

For peer review only - http://bmjopen.bmj.com/site/about/guidelines.xhtml

50. sexual abuse.tw.
51. [(partner or relationship or wom$n or domestic or spous*) adj4 (abus* or violen* or victimi* or batter*)].mp.
52. dating violence.tw.
53. sexual violence.tw.
54. rape.tw.
55. prevalence.tw.
56. cross-sectional stud$.mp. [mp = title, original title, abstract, name of substance word, subject heading word]
57. survey.mp. [mp = title, original title, abstract, name of substance word, subject heading word]
58. health survey$.mp. [mp = title, original title, abstract, name of substance word, subject heading word]
59. or/42–54
60. or/55–58
61. 59 and 60
62. 61 not 41
63. 62 and (21 or 34)

References

1. García-Moreno, C. and H. Stöckl, *Protection of sexual and reproductive health rights: Addressing violence against women.* International Journal of Gynecology & Obstetrics, 2009. **106**(2): p. 144-147.
2. Devries, K., et al., *The global prevalence of intimate partner violence against women.* Science, 2013. **340**(6140): p. 1527-1528.
3. WHO, *Global and regional estimates of violence against women: prevalence and health effects of intimate partner violence and non-partner sexual violence*. 2013: World Health Organization.
4. Stöckl, H., et al., *The global prevalence of intimate partner homicide: a systematic review.* The Lancet, 2013. **382**(9895): p. 859-865.
5. Abrahams, N., et al., *Worldwide prevalence of non-partner sexual violence: a systematic review.* The Lancet, 2014. **383**(9929): p. 1648-1654.
6. Moher, D., et al., *PRISMA statement.* Epidemiology, 2011. **22**(1): p. 128.
7. UN Department of Economic Social Affairs, P.D., *World Population Prospects: The 2017 Revision. Key Findings and Advance Tables. Working paper no. ESA/P/WP/248.* 2017.
8. WHO, *Global plan of action: health systems address violence against women and girls*. 2016, World Health Organization.

For review only

For peer review only - http://bmjopen.bmj.com/site/about/guidelines.xhtml

PRISMA-P 2015 Checklist

This checklist has been adapted for use with systematic review protocol submissions to BioMed Central journals from Table 3 in Moher D et al: Preferred reporting items for systematic review and meta-analysis protocols (PRISMA-P) 2015 statement. *Systematic Reviews* 2015 **4**:1

An Editorial from the Editors-in-Chief of *Systematic Reviews* details why this checklist was adapted - **Moher D, Stewart L & Shekelle P:** Implementing PRISMA-P: recommendations for prospective authors. *Systematic Reviews* 2016 **5**:15

Section/topic	#	Checklist item	Information reported		Line number(s)
			Yes	No	
**ADMINISTRATIVE INFORMATION**					
**Title**					
Identification	1a	Identify the report as a protocol of a systematic review	☒	☐	72
Update	1b	If the protocol is for an update of a previous systematic review, identify as such	☒	☐	93, 154
**Registration**	2	If registered, provide the name of the registry (e.g., PROSPERO) and registration number in the Abstract	☒	☐	162
**Authors**					
Contact	3a	Provide name, institutional affiliation, and e-mail address of all protocol authors; provide physical mailing address of corresponding author	☒	☐	75
Contributions	3b	Describe contributions of protocol authors and identify the guarantor of the review	☒	☐	373
**Amendments**	4	If the protocol represents an amendment of a previously completed or published protocol, identify as such and list changes; otherwise, state plan for documenting important protocol amendments	☒	☐	155
**Support**					
Sources	5a	Indicate sources of financial or other support for the review	☒	☐	367
Sponsor	5b	Provide name for the review funder and/or sponsor	☒	☐	367
Role of sponsor/funder	5c	Describe roles of funder(s), sponsor(s), and/or institution(s), if any, in developing the protocol	☒	☐	367
**INTRODUCTION**					
**Rationale**	6	Describe the rationale for the review in the context of what is already known	☒	☐	126

For peer review only - http://bmjopen.bmj.com/site/about/guidelines.xhtml

BioMed Central The Open Access Publisher

Section/topic	#	Checklist item	Information reported		Line number(s)
			Yes	No	
**Objectives**	7	Provide an explicit statement of the question(s) the review will address with reference to participants, interventions, comparators, and outcomes (PICO)	☒	☐	170
**METHODS**					
**Eligibility criteria**	8	Specify the study characteristics (e.g., PICO, study design, setting, time frame) and report characteristics (e.g., years considered, language, publication status) to be used as criteria for eligibility for the review	☒	☐	185
**Information sources**	9	Describe all intended information sources (e.g., electronic databases, contact with study authors, trial registers, or other grey literature sources) with planned dates of coverage	☒	☐	154
**Search strategy**	10	Present draft of search strategy to be used for at least one electronic database, including planned limits, such that it could be repeated	☒	☐	402
STUDY RECORDS					
Data management	11a	Describe the mechanism(s) that will be used to manage records and data throughout the review	☒	☐	248
Selection process	11b	State the process that will be used for selecting studies (e.g., two independent reviewers) through each phase of the review (i.e., screening, eligibility, and inclusion in meta-analysis)	☒	☐	248
Data collection process	11c	Describe planned method of extracting data from reports (e.g., piloting forms, done independently, in duplicate), any processes for obtaining and confirming data from investigators	☒	☐	248
**Data items**	12	List and define all variables for which data will be sought (e.g., PICO items, funding sources), any pre-planned data assumptions and simplifications	☒	☐	253
**Outcomes and prioritization**	13	List and define all outcomes for which data will be sought, including prioritization of main and additional outcomes, with rationale	☒	☐	253
**Risk of bias in individual studies**	14	Describe anticipated methods for assessing risk of bias of individual studies, including whether this will be done at the outcome or study level, or both; state how this information will be used in data synthesis	☒	☐	274
DATA					
**Synthesis**	15a	Describe criteria under which study data will be quantitatively synthesized	☒	☐	294
	15b	If data are appropriate for quantitative synthesis, describe planned summary measures, methods of handling data, and methods of combining data from studies, including any planned exploration of consistency (e.g., I^2, Kendall's tau)	☒	☐	294
	15c	Describe any proposed additional analyses (e.g., sensitivity or subgroup analyses, meta-regression)	☒	☐	294
	15d	If quantitative synthesis is not appropriate, describe the type of summary planned	☒	☐	Not applicable

For peer review only

BioMed Central The Open Access Publisher

Section/topic	#	Checklist item	Information reported: Yes	Information reported: No	Line number(s)
**Meta-bias(es)**	16	Specify any planned assessment of meta-bias(es) (e.g., publication bias across studies, selective reporting within studies)	☒	☐	294
**Confidence in cumulative evidence**	17	Describe how the strength of the body of evidence will be assessed (e.g., GRADE)	☒	☐	294s

For peer review only

BioMed Central
The Open Access Publisher

BMJ Open

Physical, sexual and psychological intimate partner violence and non-partner sexual violence against women and girls: a systematic review protocol for producing global, regional and country estimates

Journal:	*BMJ Open*
Manuscript ID	bmjopen-2020-045574.R1
Article Type:	Protocol
Date Submitted by the Author:	27-Jan-2021
Complete List of Authors:	Stöckl, Heidi; LSHTM, Global Health and Development<br>Sardinha, Lynnmarie; World Health Organization, Department of Reproductive Health and Research<br>Meyer, Sarah; World Health Organization, Department of Sexual and Reproductive Health and Research<br>Maheu-Giroux, Mathieu; McGill University, Epidemiology and Biostatistics<br>Garcia-Moreno, Claudia; World Health Organization
**Primary Subject Heading**:	Global health
Secondary Subject Heading:	Public health, Epidemiology
Keywords:	PUBLIC HEALTH, EPIDEMIOLOGY, STATISTICS & RESEARCH METHODS

SCHOLARONE™
Manuscripts

BMJ

I, the Submitting Author has the right to grant and does grant on behalf of all authors of the Work (as defined in the below author licence), an exclusive licence and/or a non-exclusive licence for contributions from authors who are: i) UK Crown employees; ii) where BMJ has agreed a CC-BY licence shall apply, and/or iii) in accordance with the terms applicable for US Federal Government officers or employees acting as part of their official duties; on a worldwide, perpetual, irrevocable, royalty-free basis to BMJ Publishing Group Ltd ("BMJ") its licensees and where the relevant Journal is co-owned by BMJ to the co-owners of the Journal, to publish the Work in this journal and any other BMJ products and to exploit all rights, as set out in our licence.

The Submitting Author accepts and understands that any supply made under these terms is made by BMJ to the Submitting Author unless you are acting as an employee on behalf of your employer or a postgraduate student of an affiliated institution which is paying any applicable article publishing charge ("APC") for Open Access articles. Where the Submitting Author wishes to make the Work available on an Open Access basis (and intends to pay the relevant APC), the terms of reuse of such Open Access shall be governed by a Creative Commons licence – details of these licences and which Creative Commons licence will apply to this Work are set out in our licence referred to above.

Other than as permitted in any relevant BMJ Author's Self Archiving Policies, I confirm this Work has not been accepted for publication elsewhere, is not being considered for publication elsewhere and does not duplicate material already published. I confirm all authors consent to publication of this Work and authorise the granting of this licence.

For peer review only

Physical, sexual and psychological intimate partner violence and non-partner sexual violence against women and girls: a systematic review protocol for producing global, regional and country estimates

Heidi Stöckl, Gender Violence & Health Centre, London School of Hygiene and Tropical Medicine, London, UK. Heidi.stoeckl@lshtm.ac.uk

LynnMarie Sardinha, Department of Reproductive Health and Research, World Health Organization, Geneva, Switzerland. sardinial@who.int

Sarah Meyer, Department of Reproductive Health and Research, World Health Organization, Geneva, Switzerland. smeyer@who.int

Mathieu Maheu-Giroux, Department of Epidemiology, Biostatistics, and Occupational Health, School of Population and Global Health, McGill University, Montréal, Canada. mathieu.maheu-giroux@mcgill.ca

Claudia García Moreno*, Department of Reproductive Health and Research, World Health Organization, Avenue Appia 20, 1211 Geneva, Geneva, Switzerland. garciamorenoc@who.int

* corresponding author

For peer review only - http://bmjopen.bmj.com/site/about/guidelines.xhtml

Abstract

Introduction: In 2013, the World Health Organisation (WHO) published the first global and regional estimates on physical and sexual intimate partner violence (IPV) and non-partner sexual violence (NPSV) based on a systematic review of population-based prevalence studies. In this protocol, we describe a new systematic review for the production of updated estimates for IPV and NPSV for global monitoring of violence against women, including providing the baseline for measuring Sustainable Development Goal to eliminate all forms of violence against women and girls.

Methods and analysis: The systematic review will update and extend the previous search for population-based surveys (either nationally or sub-nationally representative) conducted among women aged 15+ years that measured the prevalence of physical, sexual, psychological and physical and/or sexual IPV, NPSV or sexual violence by any perpetrator up to December 2019. Data will be extracted separately for all age groups, setting (urban/rural), partnership status (currently partnered/ever-partnered/all women), and recall period (lifetime prevalence/past 12 months; for IPV). Studies will be identified from electronic searches of online databases of EMBASE, MEDLINE, Global Health and PsycInfo. A search of national statistics office homepages will be conducted for each country to identify reports on population-based, national or sub-national studies that include data on IPV or NPSV published outside academic journals. Two reviewers will be involved in quality assessment and data extraction of the review. The review is planned to be updated on a continuous basis. All findings will undergo a country consultation process. It has been registered at Prospero: CRD42017054100

Ethics and dissemination: Formal ethical approval is not required, as primary data will not be collected. This systematic review will provide a basis and a follow-up tool for global monitoring of the Sustainable Development Goal Target 5.2 on the elimination of all forms of violence against women and girls.

Strength and limitations

- Gold-standard systematic review process followed by country consultation process to verify global and country estimates on intimate partner violence and non-partner sexual violence
- Multiple international technical advisory meetings to discuss the search procedures, data extraction and analysis plans of the estimates
- Consensus on definitions and comparable measurements of physical and intimate partner violence
- Challenges in the comparability of data on non-partner sexual violence measurements due to a lack of global definitions and measurement tools

Keywords: Violence against women, intimate partner violence, spousal violence, non-partner sexual violence, sexual violence, psychological abuse, psychological partner violence, systematic review, prevalence

For peer review only - http://bmjopen.bmj.com/site/about/guidelines.xhtml

Physical, sexual and psychological intimate partner violence and non-partner sexual violence against women and girls: a systematic review protocol for producing global, regional and country estimates

Background

Intimate partner violence and non-partner sexual violence are well recognized as human rights violations and public health problems [1]. In 2013, the World Health Organisation, the London School of Hygiene and Tropical Medicine, and the South African Medical Research Council conducted three systematic reviews to estimate the worldwide prevalence of intimate partner violence, non-partner sexual violence and intimate partner homicide. They estimated that one in three women experienced physical and/or sexual intimate partner violence or non-partner sexual violence in their lifetime; among all women murdered, 38.6 percent were murdered by their intimate partner [2-5]. The review further established associations between intimate partner violence and numerous adverse health outcomes, finding that women experiencing intimate partner violence were twice as likely to report depressive symptoms, nearly twice as likely to have alcohol use disorders, 16% more likely to have a low birthweight baby, and 1.5 times more likely to acquire HIV or another STI, compared to women who had not experienced intimate partner violence [3]. Similar associations were found for non-partner sexual violence and depression and alcohol use disorders [3, 5].

The growing recognition of the high prevalence and significant health and other impacts of intimate partner violence and non-partner sexual violence has contributed to the inclusion of 'the elimination of all forms of violence against women' in the 2030 Agenda for Sustainable Development. The Sustainable Development Goal (SDG) Target 5.2 on the elimination of all forms of violence against all women and girls in the public and private spheres, is monitored using the following indicators:

> 5.2.1: Proportion of ever-partnered women and girls aged 15 years and older subjected to physical, sexual or psychological violence by a current or former intimate partner in the previous 12 months, by form of violence and by age

> 5.2.2: Proportion of women and girls aged 15 years and older subjected to sexual violence by persons other than an intimate partner in the previous 12 months, by age and place of occurrence.

This article presents the protocol of the systematic review to generate the baseline data for producing estimates that will contribute to the global monitoring of violence against women, including for the SDG process.

Methods/Design

This review builds on and updates the methodology used for the previous systematic reviews on the global prevalence of intimate partner violence and non-partner sexual violence. It will be expanded to include evidence from studies directly received from national statistics offices, the Demographic and Health Surveys and those identified through additional internet searches. A data extraction form and quality assessment form used for the 2013 prevalence review data extraction to collate data from selected studies will also be adapted and expanded to include psychological intimate partner

For peer review only - http://bmjopen.bmj.com/site/about/guidelines.xhtml

violence, non-partner sexual violence and sexual violence by any partner. The Preferred Reporting Items for Systematic Review and Meta-Analysis (PRISMA) guidelines are used as the template for reporting the present review. For the present protocol, the PRISMA statement for Protocols (PRISMA-P) was used for its reporting (see online Appendix 1) [6]. This review was registered in the PROSPERO International Prospective Register of systematic reviews, registration number CRD42017054100 on the 2nd of January 2017.

Aims of the review

To collect all available data on the prevalence of physical and/or sexual intimate partner violence, psychological violence by an intimate partner, non-partner sexual violence and sexual violence by anyone, which can be used to measure advances in addressing violence against women and girls.

Specific review questions are:

1. What is the prevalence of physical and/or sexual intimate partner violence in women 15 years and older, lifetime and in the last 12 months?
2. What is the prevalence of physical intimate partner violence only, lifetime and in the last 12 months?
3. What is the prevalence of sexual intimate partner violence only, lifetime and in the last 12 months?
4. What is the prevalence of psychological intimate partner violence, lifetime and in the last 12 months and how was it measured?
5. What is the prevalence of sexual violence by perpetrator(s) other than a partner only, lifetime and in the last 12 months?
6. What is the prevalence of sexual violence by any perpetrator, lifetime and in the last 12 months?
7. How does the prevalence of intimate partner violence and non-partner sexual violence vary by age group, rural and urban settings over time, lifetime and in the last 12 months?

Criteria for considering studies for this review

Inclusion

- Type of studies: Nationally or sub-nationally representative population-based studies (cross-sectional or cohort studies).
- Type of participants: Studies of women aged 15+.
- Type of outcome: Studies measuring the prevalence of psychological, physical, sexual and physical and/or sexual intimate partner violence, non-partner sexual violence and/or sexual violence by any perpetrator (or studies providing enough data to allow computing these estimates if not directly calculated or reported).
- Only acts based measures of psychological, physical and/or sexual intimate partner violence, non-partner violence and sexual violence by any partner will be included. The authors note that there is convergence on the definitions and standardized measures of physical and sexual intimate partner violence across the world. Psychological intimate partner violence and non-partner sexual violence, however, are less well defined internationally and this systematic review therefore uses the authors' definition when extracting these data and note the different definitions used and the diversity of acts covered to define them.

For peer review only - http://bmjopen.bmj.com/site/about/guidelines.xhtml

Exclusion

- Type of studies: case reports, case series, letters, reviews, policy reports, commentaries, editorials, and administrative data including police statistics on reported crimes
- Types of participants: studies in subgroups of participants that might not be generalizable to the whole population, such as clinical, school or prison samples or studies among pregnant women, same sex partners, or police statistics of reported crimes.
- Outcome type: Estimates that combine prevalence of physical and/or sexual intimate partner violence with other forms of violence, e.g. psychological abuse or studies that did not use a "gold-standard" measure of intimate partner violence defined here as measures asking about a range of specific acts of experience of intimate partner violence because these avoid participants' subjective classification of experiences as "violence" or not.
- Minimum reporting criteria: Studies not giving sufficient detail for extrapolation or imputation, e.g. concrete sample size, confidence intervals or standard error.
- Duplicate reports and/or publications of the same data: the less comprehensive/complete and up-to-date version will be excluded if the same data is reported on. Studies of different years or from different regions identified will all be captured.
- Results will only be extracted if they are representative for a single country, province or town or a geographically restricted area within a country.

Search strategy for identifying relevant studies

A comprehensive and exhaustive search of the Ovid based databases Medline, Embase, Global Health and PsychInfo will be conducted on a regular basis, capturing the years 1989 to 2018 and thereafter to identify all relevant articles that contain data on the prevalence of psychological, physical and/or sexual intimate partner violence and non-partner sexual violence and sexual violence by any perpetrator regardless of the language of publication. The search strategy of relevant terms is detailed in Table 1. Both text words and medical subject heading terms will be used. This is a global study. Any study that is population-based and fits the inclusion criteria will be included and no restrictions in terms of country names or languages will be applied.

Searching of other data sources

Due to the increasing number of violence against women surveys conducted by local and national governments and the regular waves of the Demographic and Health Surveys with a dedicated module on 'domestic violence' which includes intimate partner violence and non-partner sexual violence, manual searches will also be conducted in DHS and other survey reports in a consistent manner in addition to signing up for the regular DHS updates [7, 8]. We will search the webpages of governmental statistical and/or other offices of each individual country for reports that include data on the prevalence of intimate partner violence or non-partner sexual violence. Each report will be traced back to its source to ensure they meet the inclusion criteria, e.g national representative survey on violence against women. Data would be excluded if it is administrative data, including police statistics on reported crimes, as these represent only the sub-set of few women who formally report violence and therefore are known to greatly underestimate the 'true' population prevalence.

Selection of studies for inclusion in the review

Two independent reviewers will use Endnote X7 to independently identify articles and sequentially screen their titles and abstracts for eligibility. Full-texts of articles deemed potentially eligible will be

For peer review only - http://bmjopen.bmj.com/site/about/guidelines.xhtml

retrieved. Further, two independent reviewers will independently assess the full-text of each study for eligibility, and consensually retain studies to be included. Dr García-Moreno will resolve disagreements. A screening guide clearly stating the inclusion and exclusion criteria will be used to ensure that the selection criteria are reliably applied by two review authors. Eligible studies in languages other than English or French will be translated using native speakers or, if unavailable, Google Translate and considered for inclusion.

Data extraction and management

Data will be extracted into an excel sheet independently by two individual researchers and divergences will be checked by a third extractor. In addition, 20% of the data entries will be checked by a third reviewer. Data extraction will furthermore be checked through cross-tabulation of similar fields before data analysis. Data extraction fields will include:

1. Study identifier: Study title, author and publication year
2. Geographical information: Region, country and iso3 codes.
3. Study characteristics: year of beginning of data collection and year of end of data collection, study design (cross-sectional, longitudinal), setting (urban, rural, sub-national, national).
4. Population characteristics: Denominator information (All women sampled, ever-partnered/ ever-married women or currently partnered/currently-married women), and age range of the sample.
5. Information on violence underlying indicator: type of violence (physical, sexual, emotional/psychological, physical and/or sexual), type of violence by perpetrator (physical intimate partner violence, sexual intimate partner violence, psychological intimate partner violence, physical and/or sexual intimate partner violence, sexual violence non-partner, any sexual violence),
6. Who was asked about violence (all women versus ever partnered vs currently partnered) , asked about intimate partner violence by current partner (current partner only or any partner), asked about violence by spouse (violence by spouse only versus violence by a spouse or partner), timing of violence (past year, past two years, ever), severe physical or sexual violence or not (definition by study).
7. Estimate: prevalence point estimate of specific form of violence, type of confidence interval, lower and upper confidence interval, standard error, denominator, numerator.
8. Key quality indicators: Study specified interviewer training on administering questions on violence against women, specialized violence against women survey or a module in a larger survey and whether study was national or subnational.

The quality of studies will be based on the following criteria, and these will be used as adjustment factors in the proposed analyses:

1. whether the study was national or sub-national, because prevalence may differ in areas within a country
2. the type and time of violence measured (whether intimate partner violence was operationalised as physical only, sexual only, past-year only, or severe only) because the prevalence of these forms of violence is lower than the combined lifetime experience of physical and/or sexual intimate partner violence.
3. Whether studies reported that interviewers were trained to ask about violence in a sensitive way as this increases participants willingness to disclose
4. whether studies were specifically designed to measure intimate partner violence or sexual violence prevalence

For peer review only - http://bmjopen.bmj.com/site/about/guidelines.xhtml

5. if studies included only currently-partnered women versus ever-partnered women, as estimates of intimate partner violence exposure in currently partnered women are likely to be lower than in ever-partnered women, particularly for lifetime prevalence, as they would not include women who have been in previously abusive relationships but not in a relationship at the time of the survey
6. if questions pertained to violence from the current partner and/or most recent partner only or from any partner.
7. if questions pertained to violence from a spouse only or from any intimate partner.

Proposed data analysis

The data from this systematic review will be used to estimate the national, global and regional prevalence of physical and/or sexual intimate partner violence, in the past 12 months and lifetime for women aged 15 years and above. For non-partner sexual violence and any sexual violence only global and regional estimates for lifetime prevalence will be produced.

For the pre-processing stage, in what we assume will be rare instances, where study's authors do not report information on the survey sample size, it will be estimated from the standard error or confidence intervals. If both are missing we will conservatively impute sample sizes corresponding to the lowest tercile of the overall distribution of survey samples sizes. If the overall survey sample size of a specific study is available but not the age-specific denominators of the prevalence estimates, we will impute them by distributing this overall sample size proportionally to the age-specific size of the female population reported in the *United Nations World Population Prospect [9]*.

For the analysis stage we will use a Bayesian meta-regression framework. This multilevel modelling approach will use survey-, country-, and region-specific random effects to pool observations from different sources and improve accuracy of estimates by drawing on information from across units. The chosen model structure is based on similar meta-regressions of health indicators [10-19]. Regions will be defined based on the *Global Burden of Diseases* (GBD) classification. Regions will groups countries in 21 mutually exclusive regions, which are situated in seven broad regions, based on the similarities of their epidemiological profiles [3, 20]. Both nationally and sub-nationally representative studies will be included. We will assume for the latter that they provide estimates that could inherently be more variable than nationally representative studies. The advantage of the proposed multilevel modelling approach is that it will allow us to pool observations together from different sources and to "*borrow strength*" across units. In case of a country with only one sub-national survey with a small sample size, for example, an empirical observation from a similar country in the same region can improve its prevalence estimate's accuracy and precision. We will also use pooling, the sharing of information between observations to improve the calculation of global estimates. Through the use of multilevel models, this will be determined empirically by the data and not arbitrarily by the user [21].

Additionally, this Bayesian regression model will consider heterogeneous age groups (using an age-standardizing approach), account for country-specific age and time trends (using splines), and adjust for key survey differences (denominator, type of violence, etc. – depending on the outcomes) through covariate modelling (with the adjustment calculated outside of the main model to avoid compositional bias). A joint model will be estimated for lifetime and past-year intimate partner violence, imposing the constraint that all age-specific past-year intimate partner violence estimates (for a particular country, at a particular time) should be lower or equal to those estimated for lifetime intimate partner violence.

For peer review only - http://bmjopen.bmj.com/site/about/guidelines.xhtml

For peer review only

For peer review only - http://bmjopen.bmj.com/site/about/guidelines.xhtml

Country consultation process

Estimates generated in this review will undergo a country consultation process, as endorsed by the WHO Executive Board in 2001 through resolution (EB.107.R8) "to ensure that each Member State is consulted on the best data to be used" for international estimation and reporting purposes[22]. In our particular case, the country consultation process was designed to enable countries to review information gathered from secondary data sources (i.e. surveys/studies) that met the inclusion criteria; ensure the inclusion of any additional surveys/studies that meet these inclusion criteria, but which may not have been previously identified; and familiarize Member States with the statistical modelling approach used to derive the country, regional and global estimates. Focal points nominated by the countries received a summary of statistical methods, translated into all six United Nation official languages and their country profile for their critical review and feedback. The country profile outlined available data sources for the estimates, population-based surveys/studies that were excluded due to not meeting the inclusion criteria, covariates for adjustment, model fits and modelled national estimates. The country consultation process also allowed countries to suggest additional surveys/studies for review, provide previously missed data and reports from unpublished surveys/studies to be reviewed and to express their interest in conducting dedicated violence against women surveys in their countries.

Patient and public involvement

No patient involved.

Discussion

This systematic review will provide critical data for the global monitoring of progress to eliminate violence against women. It will help to track the effectiveness of efforts made by governments over the coming decade in preventing and addressing intimate partner violence and non-partner sexual violence. The findings will directly feed into the global monitoring, including of the SDG Target 5.2 indicators.

Over the past years, governments have made commitments to increase the collection of data and research on violence against women, for example in the WHO Global Plan of Action [23], to strengthen the role of the health system in addressing violence, in particular against women and against children. This includes conducting population-based studies to measure the prevalence and nature of violence against women, in particular intimate partner violence and sexual violence by non-partners. This is contributing to an expanding and robust evidence base that will allow, tracking of changes over time. This systematic review will also contribute to the identification of challenges with the instruments and measures being used, especially for the forms of violence for which is there is less international agreement on measurement - psychological intimate partner violence and non-partner violence. It will also identify issues with how data is being reported.

The goal of this review and its future iterations is to contribute key epidemiological information on the magnitude and burden of violence faced by women around the world and how these prevalence patterns may differ by country, region, age, and rural/urban contexts. These data should inform the development and implementation of effective policies and programmes to prevent and respond to violence against women.

The estimates based on the findings of this study will be used by governments, published in WHO/UN reports and as scientific papers in a peer-reviewed journals, and disseminated at relevant fora. The review will be updated regularly and the estimates will be updated approximately every five years to continue to monitor progress made in addressing violence against women.

For peer review only - http://bmjopen.bmj.com/site/about/guidelines.xhtml

List of abbreviations

PRISMA: Preferred Reporting Items for Systematic Review and Meta-Analysis

TAG: Technical Advisory Group

For peer review only

For peer review only - http://bmjopen.bmj.com/site/about/guidelines.xhtml

Review status

Data extraction is ongoing as several key data points are still missing, especially on non-partner sexual violence. Existing extracted data is also currently undergoing a country consultation process, which is also adding new estimates. The methods for data analysis and for elaboration of the estimates have been tested and discussed by a technical advisory group (TAG) established by WHO and composed of independent experts. The TAG advises the UN Inter-agency Working Group on VAW data and estimates convened by WHO.

Declarations

Consent for publication: We have mostly used data publicly available or received consent for publication from national Statistical Offices for data missing in reports.

Ethics approval and consent to participate

Not applicable

Availability of data and materials

This is a study protocol. The data used for the systematic review will also be made publicly available once the systematic review will be published.

Competing interests

The authors declare that they have no competing interests

Funding

This work received funding from the UNDP-UNFPA-UNICEF-WHO-World Bank Special Programme of Research, Development and Research Training in Human Reproduction (HRP), a cosponsored programme executed by the World Health Organization (WHO). It also received funding from the Department for international Development, through the UN Women-WHO Joint Programme on Strengthening Data Collection. The funder had no role in the design of the review. Grand number: Not applicable.

Authors' contributions

Heidi Stöckl adapted the original search strategy and study design to align it with the discussions on global monitoring of VAW, including measurement of the indicators related to SDG Target 5.2. and developed the inclusion of psychological abuse and any sexual violence into the search and analysis, expanded the search strategy to capture data and reports outside the peer-reviewed literature, participated in deciding on the analysis strategy, and drafted the current manuscript.

LynnMarie Sardinha inputted into the adapted search strategy, study design and data extraction sheet, refined inclusion and exclusion criteria and quality indicators and has been involved in all decisions regarding the current search protocol and this manuscript.

Mathieu Maheu-Giroux contributed to the establishment the list of key covariates and study characteristics to collect, methods to check the database for integrity and consistency, and conceived the data analysis plan.

For peer review only - http://bmjopen.bmj.com/site/about/guidelines.xhtml

Sarah Rachel Meyer inputted into the adapted search strategy, study design and data extraction sheet, refined inclusion and exclusion criteria and quality indicators and has been involved in all decisions regarding the current search protocol and this manuscript.

Claudia García-Moreno co-conceived the original search strategy and study design, inputted into the data extraction sheet, inclusion and exclusion criteria and quality indicators, has been involved in all decisions regarding the current search protocol and this manuscript and provides technical oversight to the whole project. She is the guarantor of the review.

All authors read and approved the final manuscript.

Acknowledgements

This database updates and builds on an earlier similar effort for the WHO, 2013 estimates on the prevalence of IPV and NPSV which was led by Charlotte Watts at the LSHTM and Claudia García-Moreno of WHO. Karen Devries designed and oversaw the earlier systematic searches for intimate partner violence and Naeema Abrahams of the South African Medical Research Council for non-partner violence.

Authors should obtain permission to acknowledge from all those mentioned in the Acknowledgements section.

review only

For peer review only - http://bmjopen.bmj.com/site/about/guidelines.xhtml

Table 1: Search terms and strategy for the Ovid based databases Medline, PsychInfo, Embase and Global Health

1. meta.ab.
2. synthesis.ab.
3. literature.ab.
4. published.ab.
5. extraction.ab.
6. search.ab.
7. medline.ab.
8. selection.ab.
9. sources.ab.
10. trials.ab.
11. review.ab.
12. articles.ab.
13. reviewed.ab.
14. english.ab.
15. language.ab.
16. randomized.hw.
17. trials.hw.
18. controlled.hw.
19. meta-analysis.pt.
20. review.pt.
21. or/1–20
22. epidemiologic studies/
23. exp case control studies/
24. exp cohort studies/
25. case control.tw.
26. (cohort adj (study or studies)).tw.
27. cohort analy$.tw.
28. (follow up adj (study or studies)).tw.
29. (observational adj (study or studies)).tw.
30. longitudinal.tw.
31. retrospective.tw.
32. cross sectional studies.tw.
33. cross sectional studies/
34. or/22–33
35. Animals/
36. Humans/
37. 35 not (35 and 36)
38. comment.pt.
39. letter.pt.
40. editorial.pt.
41. or/37–40
42. domestic violence/ or partner violence/ or spouse abuse/ or spouse violence/ or domestic abuse/ or partner abuse.mp. [mp = title, original title, abstract, name of substance word, subject heading word]
43. *battered women/
44. (intimate adj4 partner adj4 violence).tw.
45. (intimate adj4 partner adj4 abuse).tw.
46. (intimate adj4 partner adj4 victimi*).tw.
47. domestic abuse.tw.
48. spou$ abuse.tw.
49. dating violence.tw.

For peer review only

For peer review only - http://bmjopen.bmj.com/site/about/guidelines.xhtml

50. sexual abuse.tw.
51. [(partner or relationship or wom$n or domestic or spous*) adj4 (abus* or violen* or victimi* or batter*)].mp.
52. dating violence.tw.
53. sexual violence.tw.
54. rape.tw.
55. prevalence.tw.
56. cross-sectional stud$.mp. [mp = title, original title, abstract, name of substance word, subject heading word]
57. survey.mp. [mp = title, original title, abstract, name of substance word, subject heading word]
58. health survey$.mp. [mp = title, original title, abstract, name of substance word, subject heading word]
59. or/42–54
60. or/55–58
61. 59 and 60
62. 61 not 41
63. 62 and (21 or 34)

For review only

References

1. García-Moreno, C. and H. Stöckl, *Protection of sexual and reproductive health rights: Addressing violence against women.* International Journal of Gynecology & Obstetrics, 2009. **106**(2): p. 144-147.
2. Devries, K., et al., *The global prevalence of intimate partner violence against women.* Science, 2013. **340**(6140): p. 1527-1528.
3. WHO, *Global and regional estimates of violence against women: prevalence and health effects of intimate partner violence and non-partner sexual violence*. 2013: World Health Organization.
4. Stöckl, H., et al., *The global prevalence of intimate partner homicide: a systematic review.* The Lancet, 2013. **382**(9895): p. 859-865.
5. Abrahams, N., et al., *Worldwide prevalence of non-partner sexual violence: a systematic review.* The Lancet, 2014. **383**(9929): p. 1648-1654.
6. Moher, D., et al., *PRISMA statement.* Epidemiology, 2011. **22**(1): p. 128.
7. Demographic and Health Surveys. *STATcompiler The DHS Program*. 2021 [cited 2021 23.01].
8. Demographic and Health Surveys. *The DHS Program - Demographic and Health Surveys*. 2021 [cited 2021 23.01].
9. UN Department of Economic Social Affairs, P.D., *World Population Prospects: The 2017 Revision. Key Findings and Advance Tables. Working paper no. ESA/P/WP/248.* 2017.
10. Devries, K.M., et al., *Global health. The global prevalence of intimate partner violence against women.* Science, 2013. **340**(6140): p. 1527-8.
11. Finucane, M.M., et al., *Bayesian Estimation of Population-Level Trends in Measures of Health Status.* Statistical Science, 2014. **29**(1): p. 18-25.
12. Danaei, G., et al., *National, regional, and global trends in systolic blood pressure since 1980: systematic analysis of health examination surveys and epidemiological studies with 786 country-years and 5.4 million participants.* Lancet, 2011. **377**(9765): p. 568-77.

For peer review only - http://bmjopen.bmj.com/site/about/guidelines.xhtml

13. Moller, A.B., et al., *Early antenatal care visit: a systematic analysis of regional and global levels and trends of coverage from 1990 to 2013.* Lancet Glob Health, 2017. **5**(10): p. e977-e983.
14. Flaxman, A.D., T. Vos, and C.J.L. Murray, *An integrative metaregression framework for descriptive epidemiology*. 2015, Seattle: University of Washington Press.
15. Maheu-Giroux, M., et al., *Prevalence of symptoms of vaginal fistula in 19 sub-Saharan Africa countries: a meta-analysis of national household survey data.* Lancet Glob Health, 2015. **3**(5): p. e271-8.
16. Abrahams, N., et al., *Worldwide prevalence of non-partner sexual violence: a systematic review.* Lancet, 2014. **383**(9929): p. 1648-1654.
17. Say, L., et al., *Global causes of maternal death: a WHO systematic analysis.* Lancet Glob Health, 2014. **2**(6): p. e323-33.
18. Alkema, L., et al., *National, regional, and global rates and trends in contraceptive prevalence and unmet need for family planning between 1990 and 2015: a systematic and comprehensive analysis.* Lancet, 2013. **381**(9878): p. 1642-52.
19. Sedgh, G., et al., *Abortion incidence between 1990 and 2014: global, regional, and subregional levels and trends.* Lancet, 2016. **388**(10041): p. 258-67.
20. Flaxman, A., T. Vos, and C. Murray, *An integrative metaregression framework for descriptive epidemiology. Publications on global health.* Institute for Health Metrics and Evaluation Seattle WA: University of Washington Press, 2015.
21. Gelman, A. and J. Hill, *Data Analysis using Regression and Multilevel/Hierarchical Models*. 2007, New York, NY: Cambridge University Press. 625.
22. Executive Board of the World Health Organization. *Resolution: Health systems performance assessment. Geneva: WHO; 19 January 2001 (EB.107.R8).* 2001 [cited accessed 27 November 2020.
23. WHO, *Global plan of action: health systems address violence against women and girls*. 2016, World Health Organization.

For peer review only

For peer review only - http://bmjopen.bmj.com/site/about/guidelines.xhtml

PRISMA-P 2015 Checklist

This checklist has been adapted for use with systematic review protocol submissions to BioMed Central journals from Table 3 in Moher D et al: Preferred reporting items for systematic review and meta-analysis protocols (PRISMA-P) 2015 statement. *Systematic Reviews* 2015 **4**:1

An Editorial from the Editors-in-Chief of *Systematic Reviews* details why this checklist was adapted - Moher D, Stewart L & Shekelle P: Implementing PRISMA-P: recommendations for prospective authors. *Systematic Reviews* 2016 **5**:15

Section/topic	#	Checklist item	Information reported		Line number(s)
			Yes	No	
**ADMINISTRATIVE INFORMATION**					
**Title**					
Identification	1a	Identify the report as a protocol of a systematic review	☒	☐	72
Update	1b	If the protocol is for an update of a previous systematic review, identify as such	☒	☐	93, 154
**Registration**	2	If registered, provide the name of the registry (e.g., PROSPERO) and registration number in the Abstract	☒	☐	162
**Authors**					
Contact	3a	Provide name, institutional affiliation, and e-mail address of all protocol authors; provide physical mailing address of corresponding author	☒	☐	75
Contributions	3b	Describe contributions of protocol authors and identify the guarantor of the review	☒	☐	373
**Amendments**	4	If the protocol represents an amendment of a previously completed or published protocol, identify as such and list changes; otherwise, state plan for documenting important protocol amendments	☒	☐	155
**Support**					
Sources	5a	Indicate sources of financial or other support for the review	☒	☐	367
Sponsor	5b	Provide name for the review funder and/or sponsor	☒	☐	367
Role of sponsor/funder	5c	Describe roles of funder(s), sponsor(s), and/or institution(s), if any, in developing the protocol	☒	☐	367
**INTRODUCTION**					
**Rationale**	6	Describe the rationale for the review in the context of what is already known	☒	☐	126

For peer review only

BioMed Central The Open Access Publisher

Section/topic	#	Checklist item	Information reported		Line number(s)
			Yes	No	
**Objectives**	7	Provide an explicit statement of the question(s) the review will address with reference to participants, interventions, comparators, and outcomes (PICO)	☒	☐	170
**METHODS**					
**Eligibility criteria**	8	Specify the study characteristics (e.g., PICO, study design, setting, time frame) and report characteristics (e.g., years considered, language, publication status) to be used as criteria for eligibility for the review	☒	☐	185
**Information sources**	9	Describe all intended information sources (e.g., electronic databases, contact with study authors, trial registers, or other grey literature sources) with planned dates of coverage	☒	☐	154
**Search strategy**	10	Present draft of search strategy to be used for at least one electronic database, including planned limits, such that it could be repeated	☒	☐	402
STUDY RECORDS					
Data management	11a	Describe the mechanism(s) that will be used to manage records and data throughout the review	☒	☐	248
Selection process	11b	State the process that will be used for selecting studies (e.g., two independent reviewers) through each phase of the review (i.e., screening, eligibility, and inclusion in meta-analysis)	☒	☐	248
Data collection process	11c	Describe planned method of extracting data from reports (e.g., piloting forms, done independently, in duplicate), any processes for obtaining and confirming data from investigators	☒	☐	248
**Data items**	12	List and define all variables for which data will be sought (e.g., PICO items, funding sources), any pre-planned data assumptions and simplifications	☒	☐	253
**Outcomes and prioritization**	13	List and define all outcomes for which data will be sought, including prioritization of main and additional outcomes, with rationale	☒	☐	253
**Risk of bias in individual studies**	14	Describe anticipated methods for assessing risk of bias of individual studies, including whether this will be done at the outcome or study level, or both; state how this information will be used in data synthesis	☒	☐	274
DATA					
**Synthesis**	15a	Describe criteria under which study data will be quantitatively synthesized	☒	☐	294
	15b	If data are appropriate for quantitative synthesis, describe planned summary measures, methods of handling data, and methods of combining data from studies, including any planned exploration of consistency (e.g., I^2, Kendall's tau)	☒	☐	294

For peer review only

BioMed Central The Open Access Publisher

Section/topic	#	Checklist item	Information reported Yes	Information reported No	Line number(s)
	15c	Describe any proposed additional analyses (e.g., sensitivity or subgroup analyses, meta-regression)	☒	☐	294
	15d	If quantitative synthesis is not appropriate, describe the type of summary planned	☒	☐	Not applicable
**Meta-bias(es)**	16	Specify any planned assessment of meta-bias(es) (e.g., publication bias across studies, selective reporting within studies)	☒	☐	294
**Confidence in cumulative evidence**	17	Describe how the strength of the body of evidence will be assessed (e.g., GRADE)	☒	☐	294s

For peer review only

BioMed Central
The Open Access Publisher

BMJ Open

Physical, sexual and psychological intimate partner violence and non-partner sexual violence against women and girls: a systematic review protocol for producing global, regional and country estimates

Journal:	*BMJ Open*
Manuscript ID	bmjopen-2020-045574.R2
Article Type:	Protocol
Date Submitted by the Author:	17-Mar-2021
Complete List of Authors:	Stöckl, Heidi; LSHTM, Global Health and Development<br>Sardinha, Lynnmarie; World Health Organization, Department of Reproductive Health and Research<br>Maheu-Giroux, Mathieu; McGill Univ, Epidemiology and Biostatistics<br>Meyer, Sarah; World Health Organization, Department of Sexual and Reproductive Health and Research<br>Garcia-Moreno, Claudia; World Health Organization
**Primary Subject Heading**:	Global health
Secondary Subject Heading:	Public health, Epidemiology
Keywords:	PUBLIC HEALTH, EPIDEMIOLOGY, STATISTICS & RESEARCH METHODS

SCHOLARONE™
Manuscripts

I, the Submitting Author has the right to grant and does grant on behalf of all authors of the Work (as defined in the below author licence), an exclusive licence and/or a non-exclusive licence for contributions from authors who are: i) UK Crown employees; ii) where BMJ has agreed a CC-BY licence shall apply, and/or iii) in accordance with the terms applicable for US Federal Government officers or employees acting as part of their official duties; on a worldwide, perpetual, irrevocable, royalty-free basis to BMJ Publishing Group Ltd ("BMJ") its licensees and where the relevant Journal is co-owned by BMJ to the co-owners of the Journal, to publish the Work in this journal and any other BMJ products and to exploit all rights, as set out in our licence.

The Submitting Author accepts and understands that any supply made under these terms is made by BMJ to the Submitting Author unless you are acting as an employee on behalf of your employer or a postgraduate student of an affiliated institution which is paying any applicable article publishing charge ("APC") for Open Access articles. Where the Submitting Author wishes to make the Work available on an Open Access basis (and intends to pay the relevant APC), the terms of reuse of such Open Access shall be governed by a Creative Commons licence – details of these licences and which Creative Commons licence will apply to this Work are set out in our licence referred to above.

Other than as permitted in any relevant BMJ Author's Self Archiving Policies, I confirm this Work has not been accepted for publication elsewhere, is not being considered for publication elsewhere and does not duplicate material already published. I confirm all authors consent to publication of this Work and authorise the granting of this licence.

For peer review only

Physical, sexual and psychological intimate partner violence and non-partner sexual violence against women and girls: a systematic review protocol for producing global, regional and country estimates

Heidi Stöckl, Gender Violence & Health Centre, London School of Hygiene and Tropical Medicine, London, UK. Heidi.stoeckl@lshtm.ac.uk

LynnMarie Sardinha, Department of Reproductive Health and Research, World Health Organization, Geneva, Switzerland. sardinial@who.int

Sarah Meyer, Department of Reproductive Health and Research, World Health Organization, Geneva, Switzerland. smeyer@who.int

Mathieu Maheu-Giroux, Department of Epidemiology, Biostatistics, and Occupational Health, School of Population and Global Health, McGill University, Montréal, Canada. mathieu.maheu-giroux@mcgill.ca

Claudia García Moreno*, Department of Reproductive Health and Research, World Health Organization, Avenue Appia 20, 1211 Geneva, Geneva, Switzerland. garciamorenoc@who.int

* corresponding author

For peer review only - http://bmjopen.bmj.com/site/about/guidelines.xhtml

Abstract

Introduction: In 2013, the World Health Organisation (WHO) published the first global and regional estimates on physical and sexual intimate partner violence (IPV) and non-partner sexual violence (NPSV) based on a systematic review of population-based prevalence studies. In this protocol, we describe a new systematic review for the production of updated estimates for IPV and NPSV for global monitoring of violence against women, including providing the baseline for measuring Sustainable Development Goal to eliminate all forms of violence against women and girls.

Methods and analysis: The systematic review will update and extend the previous search for population-based surveys (either nationally or sub-nationally representative) conducted among women aged 15+ years that measured the prevalence of physical, sexual, psychological and physical and/or sexual IPV, NPSV or sexual violence by any perpetrator up to December 2019. Data will be extracted separately for all age groups, setting (urban/rural), partnership status (currently partnered/ever-partnered/all women), and recall period (lifetime prevalence/past 12 months; for IPV). Studies will be identified from electronic searches of online databases of EMBASE, MEDLINE, Global Health and PsycInfo. A search of national statistics office homepages will be conducted for each country to identify reports on population-based, national or sub-national studies that include data on IPV or NPSV published outside academic journals. Two reviewers will be involved in quality assessment and data extraction of the review. The review is planned to be updated on a continuous basis. All findings will undergo a country consultation process. It has been registered at Prospero: CRD42017054100

Ethics and dissemination: Formal ethical approval is not required, as primary data will not be collected. This systematic review will provide a basis and a follow-up tool for global monitoring of the Sustainable Development Goal Target 5.2 on the elimination of all forms of violence against women and girls.

Strength and limitations

- Gold-standard systematic review process followed by country consultation process to verify global and country estimates on intimate partner violence and non-partner sexual violence
- Multiple international technical advisory meetings to discuss the search procedures, data extraction and analysis plans of the estimates
- Consensus on definitions and comparable measurements of physical and intimate partner violence
- Challenges in the comparability of data on non-partner sexual violence measurements due to a lack of global definitions and measurement tools

Keywords: Violence against women, intimate partner violence, spousal violence, non-partner sexual violence, sexual violence, psychological abuse, psychological partner violence, systematic review, prevalence

For peer review only - http://bmjopen.bmj.com/site/about/guidelines.xhtml

Physical, sexual and psychological intimate partner violence and non-partner sexual violence against women and girls: a systematic review protocol for producing global, regional and country estimates

Background

Intimate partner violence and non-partner sexual violence are well recognized as human rights violations and public health problems [1]. In 2013, the World Health Organisation, the London School of Hygiene and Tropical Medicine, and the South African Medical Research Council conducted three systematic reviews to estimate the worldwide prevalence of intimate partner violence, non-partner sexual violence and intimate partner homicide. They estimated that one in three women experienced physical and/or sexual intimate partner violence or non-partner sexual violence in their lifetime; among all women murdered, 38.6 percent were murdered by their intimate partner [2-5]. The review further established associations between intimate partner violence and numerous adverse health outcomes, finding that women experiencing intimate partner violence were twice as likely to report depressive symptoms, nearly twice as likely to have alcohol use disorders, 16% more likely to have a low birthweight baby, and 1.5 times more likely to acquire HIV or another STI, compared to women who had not experienced intimate partner violence [3]. Similar associations were found for non-partner sexual violence and depression and alcohol use disorders [3, 5].

The growing recognition of the high prevalence and significant health and other impacts of intimate partner violence and non-partner sexual violence has contributed to the inclusion of 'the elimination of all forms of violence against women' in the 2030 Agenda for Sustainable Development. The Sustainable Development Goal (SDG) Target 5.2 on the elimination of all forms of violence against all women and girls in the public and private spheres, is monitored using the following indicators:

> 5.2.1: Proportion of ever-partnered women and girls aged 15 years and older subjected to physical, sexual or psychological violence by a current or former intimate partner in the previous 12 months, by form of violence and by age

> 5.2.2: Proportion of women and girls aged 15 years and older subjected to sexual violence by persons other than an intimate partner in the previous 12 months, by age and place of occurrence.

This article presents the protocol of the systematic review to generate the baseline data for producing estimates that will contribute to the global monitoring of violence against women, including for the SDG process.

Methods/Design

This review builds on and updates the methodology used for the previous systematic reviews on the global prevalence of intimate partner violence and non-partner sexual violence. It will be expanded to include evidence from studies directly received from national statistics offices, the Demographic and Health Surveys and those identified through additional internet searches. A data extraction form and quality assessment form used for the 2013 prevalence review data extraction to collate data from selected studies will also be adapted and expanded to include psychological intimate partner

For peer review only - http://bmjopen.bmj.com/site/about/guidelines.xhtml

violence, non-partner sexual violence and sexual violence by any partner. The Preferred Reporting Items for Systematic Review and Meta-Analysis (PRISMA) guidelines are used as the template for reporting the present review. For the present protocol, the PRISMA statement for Protocols (PRISMA-P) was used for its reporting (see online Appendix 1) [6]. This review was registered in the PROSPERO International Prospective Register of systematic reviews, registration number CRD42017054100 on the 2nd of January 2017.

Aims of the review

To collect all available data on the prevalence of physical and/or sexual intimate partner violence, psychological violence by an intimate partner, non-partner sexual violence and sexual violence by anyone, which can be used to measure advances in addressing violence against women and girls.

Specific review questions are:

1. What is the prevalence of physical and/or sexual intimate partner violence in women 15 years and older, lifetime and in the last 12 months?
2. What is the prevalence of physical intimate partner violence only, lifetime and in the last 12 months?
3. What is the prevalence of sexual intimate partner violence only, lifetime and in the last 12 months?
4. What is the prevalence of psychological intimate partner violence, lifetime and in the last 12 months and how was it measured?
5. What is the prevalence of sexual violence by perpetrator(s) other than a partner only, lifetime and in the last 12 months?
6. What is the prevalence of sexual violence by any perpetrator, lifetime and in the last 12 months?
7. How does the prevalence of intimate partner violence and non-partner sexual violence vary by age group, rural and urban settings over time, lifetime and in the last 12 months?

Criteria for considering studies for this review

Inclusion

- Type of studies: Nationally or sub-nationally representative population-based studies (cross-sectional or cohort studies).
- Type of participants: Studies of women aged 15+.
- Type of outcome: Studies measuring the prevalence of psychological, physical, sexual and physical and/or sexual intimate partner violence, non-partner sexual violence and/or sexual violence by any perpetrator (or studies providing enough data to allow computing these estimates if not directly calculated or reported).
- Only acts based measures of psychological, physical and/or sexual intimate partner violence, non-partner violence and sexual violence by any partner will be included. The authors note that there is convergence on the definitions and standardized measures of physical and sexual intimate partner violence across the world. Psychological intimate partner violence and non-partner sexual violence, however, are less well defined internationally and this systematic review therefore uses the authors' definition when extracting these data and note the different definitions used and the diversity of acts covered to define them.

For peer review only - http://bmjopen.bmj.com/site/about/guidelines.xhtml

Exclusion

- Type of studies: case reports, case series, letters, reviews, policy reports, commentaries, editorials, and administrative data including police statistics on reported crimes
- Types of participants: studies in subgroups of participants that might not be generalizable to the whole population, such as clinical, school or prison samples or studies among pregnant women, same sex partners, or police statistics of reported crimes.
- Outcome type: Estimates that combine prevalence of physical and/or sexual intimate partner violence with other forms of violence, e.g. psychological abuse or studies that did not use a "gold-standard" measure of intimate partner violence defined here as measures asking about a range of specific acts of experience of intimate partner violence because these avoid participants' subjective classification of experiences as "violence" or not.
- Minimum reporting criteria: Studies not giving sufficient detail for extrapolation or imputation, e.g. concrete sample size, confidence intervals or standard error.
- Duplicate reports and/or publications of the same data: the less comprehensive/complete and up-to-date version will be excluded if the same data is reported on. Studies of different years or from different regions identified will all be captured.
- Results will only be extracted if they are representative for a single country, province or town or a geographically restricted area within a country.

Search strategy for identifying relevant studies

A comprehensive and exhaustive search of the Ovid based databases Medline, Embase, Global Health and PsychInfo will be conducted on a regular basis, capturing the years 1989 to 2018 and thereafter to identify all relevant articles that contain data on the prevalence of psychological, physical and/or sexual intimate partner violence and non-partner sexual violence and sexual violence by any perpetrator regardless of the language of publication. The search strategy of relevant terms is detailed in Table 1. Both text words and medical subject heading terms will be used. This is a global study. Any study that is population-based and fits the inclusion criteria will be included and no restrictions in terms of country names or languages will be applied.

Searching of other data sources

Due to the increasing number of violence against women surveys conducted by local and national governments and the regular waves of the Demographic and Health Surveys with a dedicated module on 'domestic violence' which includes intimate partner violence and non-partner sexual violence, manual searches will also be conducted in DHS and other survey reports in a consistent manner in addition to signing up for the regular DHS updates [7, 8]. We will search the webpages of governmental statistical and/or other offices of each individual country for reports that include data on the prevalence of intimate partner violence or non-partner sexual violence. Each report will be traced back to its source to ensure they meet the inclusion criteria, e.g national representative survey on violence against women. Data would be excluded if it is administrative data, including police statistics on reported crimes, as these represent only the sub-set of few women who formally report violence and therefore are known to greatly underestimate the 'true' population prevalence.

Selection of studies for inclusion in the review

Two independent reviewers will use Endnote X7 to independently identify articles and sequentially screen their titles and abstracts for eligibility. Full-texts of articles deemed potentially eligible will be

For peer review only - http://bmjopen.bmj.com/site/about/guidelines.xhtml

retrieved. Further, two independent reviewers will independently assess the full-text of each study for eligibility, and consensually retain studies to be included. Dr García-Moreno will resolve disagreements. A screening guide clearly stating the inclusion and exclusion criteria will be used to ensure that the selection criteria are reliably applied by two review authors. Eligible studies in languages other than English or French will be translated using native speakers or, if unavailable, Google Translate and considered for inclusion.

Data extraction and management

Data will be extracted into an excel sheet independently by two individual researchers and divergences will be checked by a third extractor. In addition, 20% of the data entries will be checked by a third reviewer. Data extraction will furthermore be checked through cross-tabulation of similar fields before data analysis. Data extraction fields will include:

1. Study identifier: Study title, author and publication year
2. Geographical information: Region, country and iso3 codes.
3. Study characteristics: year of beginning of data collection and year of end of data collection, study design (cross-sectional, longitudinal), setting (urban, rural, sub-national, national).
4. Population characteristics: Denominator information (All women sampled, ever-partnered/ ever-married women or currently partnered/currently-married women), and age range of the sample.
5. Information on violence underlying indicator: type of violence (physical, sexual, emotional/psychological, physical and/or sexual), type of violence by perpetrator (physical intimate partner violence, sexual intimate partner violence, psychological intimate partner violence, physical and/or sexual intimate partner violence, sexual violence non-partner, any sexual violence),
6. Who was asked about violence (all women versus ever partnered vs currently partnered) , asked about intimate partner violence by current partner (current partner only or any partner), asked about violence by spouse (violence by spouse only versus violence by a spouse or partner), timing of violence (past year, past two years, ever), severe physical or sexual violence or not (definition by study).
7. Estimate: prevalence point estimate of specific form of violence, type of confidence interval, lower and upper confidence interval, standard error, denominator, numerator.
8. Key quality indicators: Study specified interviewer training on administering questions on violence against women, specialized violence against women survey or a module in a larger survey and whether study was national or subnational.

The quality of studies will be based on the following criteria, and these will be used as adjustment factors in the proposed analyses:

1. whether the study was national or sub-national, because prevalence may differ in areas within a country
2. the type and time of violence measured (whether intimate partner violence was operationalised as physical only, sexual only, past-year only, or severe only) because the prevalence of these forms of violence is lower than the combined lifetime experience of physical and/or sexual intimate partner violence.
3. Whether studies reported that interviewers were trained to ask about violence in a sensitive way as this increases participants willingness to disclose
4. whether studies were specifically designed to measure intimate partner violence or sexual violence prevalence

For peer review only - http://bmjopen.bmj.com/site/about/guidelines.xhtml

5. if studies included only currently-partnered women versus ever-partnered women, as estimates of intimate partner violence exposure in currently partnered women are likely to be lower than in ever-partnered women, particularly for lifetime prevalence, as they would not include women who have been in previously abusive relationships but not in a relationship at the time of the survey
6. if questions pertained to violence from the current partner and/or most recent partner only or from any partner.
7. if questions pertained to violence from a spouse only or from any intimate partner.

Proposed data analysis

The data from this systematic review will be used to estimate the national, global and regional prevalence of physical and/or sexual intimate partner violence, in the past 12 months and lifetime for women aged 15 years and above. For non-partner sexual violence and any sexual violence only global and regional estimates for lifetime prevalence will be produced.

For the pre-processing stage, in what we assume will be rare instances, where study's authors do not report information on the survey sample size, it will be estimated from the standard error or confidence intervals. If both are missing we will conservatively impute sample sizes corresponding to the lowest tercile of the overall distribution of survey samples sizes. If the overall survey sample size of a specific study is available but not the age-specific denominators of the prevalence estimates, we will impute them by distributing this overall sample size proportionally to the age-specific size of the female population reported in the *United Nations World Population Prospect [9]*.

For the analysis stage we will use a Bayesian meta-regression framework. This multilevel modelling approach will use survey-, country-, and region-specific random effects to pool observations from different sources and improve accuracy of estimates by drawing on information from across units. The chosen model structure is based on similar meta-regressions of health indicators [10-19]. Regions will be defined based on the *Global Burden of Diseases* (GBD) classification. Regions will groups countries in 21 mutually exclusive regions, which are situated in seven broad regions, based on the similarities of their epidemiological profiles [3, 20]. Both nationally and sub-nationally representative studies will be included. We will assume for the latter that they provide estimates that could inherently be more variable than nationally representative studies. The advantage of the proposed multilevel modelling approach is that it will allow us to pool observations together from different sources and to "*borrow strength*" across units. In case of a country with only one sub-national survey with a small sample size, for example, an empirical observation from a similar country in the same region can improve its prevalence estimate's accuracy and precision. We will also use pooling, the sharing of information between observations to improve the calculation of global estimates. Through the use of multilevel models, this will be determined empirically by the data and not arbitrarily by the user [21].

Additionally, this Bayesian regression model will consider heterogeneous age groups (using an age-standardizing approach), account for country-specific age and time trends (using splines), and adjust for key survey differences (denominator, type of violence, etc. – depending on the outcomes) through covariate modelling (with the adjustment calculated outside of the main model to avoid compositional bias). A joint model will be estimated for lifetime and past-year intimate partner violence, imposing the constraint that all age-specific past-year intimate partner violence estimates (for a particular country, at a particular time) should be lower or equal to those estimated for lifetime intimate partner violence.

For peer review only - http://bmjopen.bmj.com/site/about/guidelines.xhtml

Country consultation process

Estimates generated in this review will undergo a country consultation process, as endorsed by the WHO Executive Board in 2001 through resolution (EB.107.R8) "to ensure that each Member State is consulted on the best data to be used" for international estimation and reporting purposes[22]. In our particular case, the country consultation process was designed to enable countries to review information gathered from secondary data sources (i.e. surveys/studies) that met the inclusion criteria; ensure the inclusion of any additional surveys/studies that meet these inclusion criteria, but which may not have been previously identified; and familiarize Member States with the statistical modelling approach used to derive the country, regional and global estimates. Focal points nominated by the countries received a summary of statistical methods, translated into all six United Nation official languages and their country profile for their critical review and feedback. The country profile outlined available data sources for the estimates, population-based surveys/studies that were excluded due to not meeting the inclusion criteria, covariates for adjustment, model fits and modelled national estimates. The country consultation process also allowed countries to suggest additional surveys/studies for review, provide previously missed data and reports from unpublished surveys/studies to be reviewed and to express their interest in conducting dedicated violence against women surveys in their countries.

Patient and public involvement

No patient involved.

Ethics and dissemination

Formal ethical approval is not required, as primary data will not be collected. This systematic review will provide a basis and a follow-up tool for global monitoring of the Sustainable Development Goal Target 5.2 on the elimination of all forms of violence against women and girls.

Discussion

This systematic review will provide critical data for the global monitoring of progress to eliminate violence against women. It will help to track the effectiveness of efforts made by governments over the coming decade in preventing and addressing intimate partner violence and non-partner sexual violence. The findings will directly feed into the global monitoring, including of the SDG Target 5.2 indicators.

Over the past years, governments have made commitments to increase the collection of data and research on violence against women, for example in the WHO Global Plan of Action [23], to strengthen the role of the health system in addressing violence, in particular against women and against children. This includes conducting population-based studies to measure the prevalence and nature of violence against women, in particular intimate partner violence and sexual violence by non-partners. This is contributing to an expanding and robust evidence base that will allow, tracking of changes over time. This systematic review will also contribute to the identification of challenges with the instruments and measures being used, especially for the forms of violence for which is there is less international agreement on measurement - psychological intimate partner violence and non-partner violence. It will also identify issues with how data is being reported.

For peer review only - http://bmjopen.bmj.com/site/about/guidelines.xhtml

The goal of this review and its future iterations is to contribute key epidemiological information on the magnitude and burden of violence faced by women around the world and how these prevalence patterns may differ by country, region, age, and rural/urban contexts. These data should inform the development and implementation of effective policies and programmes to prevent and respond to violence against women.

The estimates based on the findings of this study will be used by governments, published in WHO/UN reports and as scientific papers in a peer-reviewed journals, and disseminated at relevant fora. The review will be updated regularly and the estimates will be updated approximately every five years to continue to monitor progress made in addressing violence against women.

List of abbreviations

PRISMA: Preferred Reporting Items for Systematic Review and Meta-Analysis

TAG: Technical Advisory Group

For peer review only

For peer review only - http://bmjopen.bmj.com/site/about/guidelines.xhtml

Review status

Data extraction is ongoing as several key data points are still missing, especially on non-partner sexual violence. Existing extracted data is also currently undergoing a country consultation process, which is also adding new estimates. The methods for data analysis and for elaboration of the estimates have been tested and discussed by a technical advisory group (TAG) established by WHO and composed of independent experts. The TAG advises the UN Inter-agency Working Group on VAW data and estimates convened by WHO.

Declarations

Consent for publication: We have mostly used data publicly available or received consent for publication from national Statistical Offices for data missing in reports.

Availability of data and materials

This is a study protocol. The data used for the systematic review will also be made publicly available once the systematic review will be published.

Competing interests

There are no competing interests for any author.

Funding

This work received funding from the Department for international Development, through the UN Women-WHO Joint Programme on Strengthening Data Collection and the UNDP-UNFPA-UNICEF-WHO-World Bank Special Programme of Research, Development and Research Training in Human Reproduction (HRP), a cosponsored programme executed by the World Health Organization (WHO). The funders had no role in the design of the review. Grand number: Not applicable.

Authors' contributions

Heidi Stöckl adapted the original search strategy and study design to align it with the discussions on global monitoring of VAW, including measurement of the indicators related to SDG Target 5.2. and developed the inclusion of psychological abuse and any sexual violence into the search and analysis, expanded the search strategy to capture data and reports outside the peer-reviewed literature, participated in deciding on the analysis strategy, and drafted the current manuscript.

LynnMarie Sardinha inputted into the adapted search strategy, study design and data extraction sheet, refined inclusion and exclusion criteria and quality indicators and has been involved in all decisions regarding the current search protocol and this manuscript.

Mathieu Maheu-Giroux contributed to the establishment the list of key covariates and study characteristics to collect, methods to check the database for integrity and consistency, and conceived the data analysis plan.

Sarah Rachel Meyer inputted into the adapted search strategy, study design and data extraction sheet, refined inclusion and exclusion criteria and quality indicators and has been involved in all decisions regarding the current search protocol and this manuscript.

For peer review only - http://bmjopen.bmj.com/site/about/guidelines.xhtml

Claudia García-Moreno co-conceived the original search strategy and study design, inputted into the data extraction sheet, inclusion and exclusion criteria and quality indicators, has been involved in all decisions regarding the current search protocol and this manuscript and provides technical oversight to the whole project. She is the guarantor of the review.

All authors read and approved the final manuscript.

Acknowledgements

This database updates and builds on an earlier similar effort for the WHO, 2013 estimates on the prevalence of IPV and NPSV which was led by Charlotte Watts at the LSHTM and Claudia García-Moreno of WHO. Karen Devries designed and oversaw the earlier systematic searches for intimate partner violence and Naeema Abrahams of the South African Medical Research Council for non-partner violence.

Authors should obtain permission to acknowledge from all those mentioned in the Acknowledgements section.

For peer review only

Table 1: Search terms and strategy for the Ovid based databases Medline, PsychInfo, Embase and Global Health

1. meta.ab.
2. synthesis.ab.
3. literature.ab.
4. published.ab.
5. extraction.ab.
6. search.ab.
7. medline.ab.
8. selection.ab.
9. sources.ab.
10. trials.ab.
11. review.ab.
12. articles.ab.
13. reviewed.ab.
14. english.ab.
15. language.ab.
16. randomized.hw.
17. trials.hw.
18. controlled.hw.
19. meta-analysis.pt.
20. review.pt.
21. or/1–20
22. epidemiologic studies/
23. exp case control studies/
24. exp cohort studies/
25. case control.tw.
26. (cohort adj (study or studies)).tw.
27. cohort analy$.tw.
28. (follow up adj (study or studies)).tw.
29. (observational adj (study or studies)).tw.
30. longitudinal.tw.
31. retrospective.tw.
32. cross sectional studies.tw.
33. cross sectional studies/
34. or/22–33
35. Animals/
36. Humans/
37. 35 not (35 and 36)
38. comment.pt.
39. letter.pt.
40. editorial.pt.
41. or/37–40
42. domestic violence/ or partner violence/ or spouse abuse/ or spouse violence/ or domestic abuse/ or partner abuse.mp. [mp = title, original title, abstract, name of substance word, subject heading word]
43. *battered women/
44. (intimate adj4 partner adj4 violence).tw.
45. (intimate adj4 partner adj4 abuse).tw.
46. (intimate adj4 partner adj4 victimi*).tw.
47. domestic abuse.tw.
48. spou$ abuse.tw.
49. dating violence.tw.

For peer review only

For peer review only - http://bmjopen.bmj.com/site/about/guidelines.xhtml

50. sexual abuse.tw.
51. [(partner or relationship or wom$n or domestic or spous*) adj4 (abus* or violen* or victimi* or batter*)].mp.
52. dating violence.tw.
53. sexual violence.tw.
54. rape.tw.
55. prevalence.tw.
56. cross-sectional stud$.mp. [mp = title, original title, abstract, name of substance word, subject heading word]
57. survey.mp. [mp = title, original title, abstract, name of substance word, subject heading word]
58. health survey$.mp. [mp = title, original title, abstract, name of substance word, subject heading word]
59. or/42–54
60. or/55–58
61. 59 and 60
62. 61 not 41
63. 62 and (21 or 34)

Ethics approval and consent to participate

Not applicable

References

1. García-Moreno, C. and H. Stöckl, *Protection of sexual and reproductive health rights: Addressing violence against women.* International Journal of Gynecology & Obstetrics, 2009. **106**(2): p. 144-147.
2. Devries, K., et al., *The global prevalence of intimate partner violence against women.* Science, 2013. **340**(6140): p. 1527-1528.
3. WHO, *Global and regional estimates of violence against women: prevalence and health effects of intimate partner violence and non-partner sexual violence*. 2013: World Health Organization.
4. Stöckl, H., et al., *The global prevalence of intimate partner homicide: a systematic review.* The Lancet, 2013. **382**(9895): p. 859-865.
5. Abrahams, N., et al., *Worldwide prevalence of non-partner sexual violence: a systematic review.* The Lancet, 2014. **383**(9929): p. 1648-1654.
6. Moher, D., et al., *PRISMA statement.* Epidemiology, 2011. **22**(1): p. 128.
7. Demographic and Health Surveys. *STATcompiler The DHS Program*. 2021 [cited 2021 23.01].
8. Demographic and Health Surveys. *The DHS Program - Demographic and Health Surveys*. 2021 [cited 2021 23.01].
9. UN Department of Economic Social Affairs, P.D., *World Population Prospects: The 2017 Revision. Key Findings and Advance Tables. Working paper no. ESA/P/WP/248.* 2017.
10. Devries, K.M., et al., *Global health. The global prevalence of intimate partner violence against women.* Science, 2013. **340**(6140): p. 1527-8.
11. Finucane, M.M., et al., *Bayesian Estimation of Population-Level Trends in Measures of Health Status.* Statistical Science, 2014. **29**(1): p. 18-25.
12. Danaei, G., et al., *National, regional, and global trends in systolic blood pressure since 1980: systematic analysis of health examination surveys and epidemiological studies with 786 country-years and 5.4 million participants.* Lancet, 2011. **377**(9765): p. 568-77.

For peer review only

13. Moller, A.B., et al., *Early antenatal care visit: a systematic analysis of regional and global levels and trends of coverage from 1990 to 2013.* Lancet Glob Health, 2017. **5**(10): p. e977-e983.
14. Flaxman, A.D., T. Vos, and C.J.L. Murray, *An integrative metaregression framework for descriptive epidemiology*. 2015, Seattle: University of Washington Press.
15. Maheu-Giroux, M., et al., *Prevalence of symptoms of vaginal fistula in 19 sub-Saharan Africa countries: a meta-analysis of national household survey data.* Lancet Glob Health, 2015. **3**(5): p. e271-8.
16. Abrahams, N., et al., *Worldwide prevalence of non-partner sexual violence: a systematic review.* Lancet, 2014. **383**(9929): p. 1648-1654.
17. Say, L., et al., *Global causes of maternal death: a WHO systematic analysis.* Lancet Glob Health, 2014. **2**(6): p. e323-33.
18. Alkema, L., et al., *National, regional, and global rates and trends in contraceptive prevalence and unmet need for family planning between 1990 and 2015: a systematic and comprehensive analysis.* Lancet, 2013. **381**(9878): p. 1642-52.
19. Sedgh, G., et al., *Abortion incidence between 1990 and 2014: global, regional, and subregional levels and trends.* Lancet, 2016. **388**(10041): p. 258-67.
20. Flaxman, A., T. Vos, and C. Murray, *An integrative metaregression framework for descriptive epidemiology. Publications on global health.* Institute for Health Metrics and Evaluation Seattle WA: University of Washington Press, 2015.
21. Gelman, A. and J. Hill, *Data Analysis using Regression and Multilevel/Hierarchical Models*. 2007, New York, NY: Cambridge University Press. 625.
22. Executive Board of the World Health Organization. *Resolution: Health systems performance assessment. Geneva: WHO; 19 January 2001 (EB.107.R8).* 2001 [cited accessed 27 November 2020.
23. WHO, *Global plan of action: health systems address violence against women and girls*. 2016, World Health Organization.

For peer review only - http://bmjopen.bmj.com/site/about/guidelines.xhtml

1

PRISMA-P 2015 Checklist

This checklist has been adapted for use with systematic review protocol submissions to BioMed Central journals from Table 3 in Moher D et al: Preferred reporting items for systematic review and meta-analysis protocols (PRISMA-P) 2015 statement. *Systematic Reviews* 2015 **4**:1

An Editorial from the Editors-in-Chief of *Systematic Reviews* details why this checklist was adapted - Moher D, Stewart L & Shekelle P: Implementing PRISMA-P: recommendations for prospective authors. *Systematic Reviews* 2016 **5**:15

Section/topic	#	Checklist item	Information reported Yes	Information reported No	Line number(s)
**ADMINISTRATIVE INFORMATION**					
**Title**					
Identification	1a	Identify the report as a protocol of a systematic review	☒	☐	72
Update	1b	If the protocol is for an update of a previous systematic review, identify as such	☒	☐	93, 154
**Registration**	2	If registered, provide the name of the registry (e.g., PROSPERO) and registration number in the Abstract	☒	☐	162
**Authors**					
Contact	3a	Provide name, institutional affiliation, and e-mail address of all protocol authors; provide physical mailing address of corresponding author	☒	☐	75
Contributions	3b	Describe contributions of protocol authors and identify the guarantor of the review	☒	☐	373
**Amendments**	4	If the protocol represents an amendment of a previously completed or published protocol, identify as such and list changes; otherwise, state plan for documenting important protocol amendments	☒	☐	155
**Support**					
Sources	5a	Indicate sources of financial or other support for the review	☒	☐	367
Sponsor	5b	Provide name for the review funder and/or sponsor	☒	☐	367
Role of sponsor/funder	5c	Describe roles of funder(s), sponsor(s), and/or institution(s), if any, in developing the protocol	☒	☐	367
**INTRODUCTION**					
**Rationale**	6	Describe the rationale for the review in the context of what is already known	☒	☐	126

BioMed Central
The Open Access Publisher

Section/topic	#	Checklist item	Information reported Yes	Information reported No	Line number(s)
**Objectives**	7	Provide an explicit statement of the question(s) the review will address with reference to participants, interventions, comparators, and outcomes (PICO)	☒	☐	170
**METHODS**					
**Eligibility criteria**	8	Specify the study characteristics (e.g., PICO, study design, setting, time frame) and report characteristics (e.g., years considered, language, publication status) to be used as criteria for eligibility for the review	☒	☐	185
**Information sources**	9	Describe all intended information sources (e.g., electronic databases, contact with study authors, trial registers, or other grey literature sources) with planned dates of coverage	☒	☐	154
**Search strategy**	10	Present draft of search strategy to be used for at least one electronic database, including planned limits, such that it could be repeated	☒	☐	402
STUDY RECORDS					
Data management	11a	Describe the mechanism(s) that will be used to manage records and data throughout the review	☒	☐	248
Selection process	11b	State the process that will be used for selecting studies (e.g., two independent reviewers) through each phase of the review (i.e., screening, eligibility, and inclusion in meta-analysis)	☒	☐	248
Data collection process	11c	Describe planned method of extracting data from reports (e.g., piloting forms, done independently, in duplicate), any processes for obtaining and confirming data from investigators	☒	☐	248
**Data items**	12	List and define all variables for which data will be sought (e.g., PICO items, funding sources), any pre-planned data assumptions and simplifications	☒	☐	253
**Outcomes and prioritization**	13	List and define all outcomes for which data will be sought, including prioritization of main and additional outcomes, with rationale	☒	☐	253
**Risk of bias in individual studies**	14	Describe anticipated methods for assessing risk of bias of individual studies, including whether this will be done at the outcome or study level, or both; state how this information will be used in data synthesis	☒	☐	274
DATA					
**Synthesis**	15a	Describe criteria under which study data will be quantitatively synthesized	☒	☐	294
	15b	If data are appropriate for quantitative synthesis, describe planned summary measures, methods of handling data, and methods of combining data from studies, including any planned exploration of consistency (e.g., I^2, Kendall's tau)	☒	☐	294

BioMed Central The Open Access Publisher

For peer review only - http://bmjopen.bmj.com/site/about/guidelines.xhtml

3

Section/topic	#	Checklist item	Information reported		Line number(s)
			Yes	No	
	15c	Describe any proposed additional analyses (e.g., sensitivity or subgroup analyses, meta-regression)	☒	☐	294
	15d	If quantitative synthesis is not appropriate, describe the type of summary planned	☒	☐	Not applicable
**Meta-bias(es)**	16	Specify any planned assessment of meta-bias(es) (e.g., publication bias across studies, selective reporting within studies)	☒	☐	294
**Confidence in cumulative evidence**	17	Describe how the strength of the body of evidence will be assessed (e.g., GRADE)	☒	☐	294s

For peer review only

BioMed Central
The Open Access Publisher
